# Association between Metabolic-Dysfunction-Associated Steatotic Liver Disease and Hepatic Cancer: Current Concepts and Future Challenges

**DOI:** 10.3390/jcm13113132

**Published:** 2024-05-27

**Authors:** Husam Bader, Saif Yamin, Hamzeh Alshahwan, Husam Farraj, Joud Maghnam, Yazan Abu Omar

**Affiliations:** 1 Department of Internal Medicine, University of Michigan, Ann Arbor, MI 48109, USA; h.bader87@gmail.com (H.B.); yazanao91@gmail.com (Y.A.O.); 2 School of Medicine, University of Jordan, Amman 11942, Jordan; alshahwanhamzeh@gmail.com; 3 Department of Internal Medicine, University of New Mexico, Albuquerque, NM 87106, USA; husam_farraj@hotmail.com; 4 School of Medicine, Al-Balqa’ Applied University, Al-Salt 19117, Jordan; jmaghnam6@gmail.com

**Keywords:** non-alcoholic fatty liver disease, metabolic-dysfunction-associated steatotic liver disease, hepatic cancer, hepatocellular carcinoma, liver fibrosis, liver fibrosis score, FIB-4 index

## Abstract

**Background:** This study systematically reviewed the association between metabolic-dysfunction-associated steatotic liver disease (MASLD) and the development of hepatic cancer. Previous research has highlighted MASLD as a predisposing condition. **Aim:** To collect recent global data on the relationship between MASLD and hepatic cancer. **Methods:** A systematic review was conducted, which included an analysis of studies on the relationship between MASLD and the incidence of hepatic cancers, focusing on the role of fibrosis and MASLD severity as predictors of cancer risk. Following standard methodological frameworks for the assessment of longitudinal studies, the review gathered information on fibrosis scores, hepatocellular carcinoma (HCC) incidence, and other types of hepatic neoplasms. **Results:** A total of 522 studies were initially identified, of which 6 studies were appropriate for the review. They collectively revealed that the stage of fibrosis in MASLD is a significant independent predictor of mortality and liver-related events, with higher fibrosis stages correlating with greater risk. Longitudinal data showed that increases in FIB-4 scores were linked to a higher risk of developing HCC and cirrhosis. MASLD was also associated with an increased risk of non-hepatic cancers such as colorectal cancer in males and breast cancer in females. The severity of MASLD was found to be a modifiable risk factor for biliary tract cancer (BTC), with the risk further amplified by diabetes. Moreover, lifestyle factors and comorbidities, such as smoking and diabetes, were identified as modifiers of cancer risk in MASLD patients. **Conclusions:** The systematic review identified the association between MASLD and an elevated risk of hepatic cancer, establishing a clear link between the severity of liver fibrosis and the incidence of HCC and other hepatic neoplasms. This supports the need for screening for hepatic cancer in patients with MASLD, particularly in the presence of advanced fibrosis or other risk-modifying factors.

## 1. Introduction

The medical community has transitioned from non-alcoholic fatty liver disease (NAFLD) to metabolic-dysfunction-associated steatotic liver disease (MASLD), which represents a spectrum of liver conditions characterized by hepatic steatosis in the absence of significant alcohol consumption [1]. However, much of the data available on NAFLD can still be widely used for MASLD [2]. As one of the most common chronic liver disorders in the developed world, MASLD encompasses a range of pathologies from simple steatosis to metabolic dysfunction-associated steatohepatitis (MASH), formerly non-alcoholic steatohepatitis (NASH) [1,3]. The increasing prevalence of MASLD is directly linked to the rising trend of metabolic syndrome constituents, including obesity, insulin resistance, and dyslipidemia, positioning MASLD as a critical public health concern with various systemic implications [4].

The progression from simple steatosis to MASH or advanced fibrosis is not guaranteed, and the course of MASLD varies among patients [5]. A particularly threatening sequela of MASLD is the evolution into cirrhosis, which can lead to severe complications and potentially hepatocellular carcinoma, the most prevalent form of primary hepatic cancer [6], often requiring liver transplantation. It is now a primary reason for liver transplants, particularly in major medical centers [7].

The pathogenetic mechanisms behind the transition from MASLD to HCC are complex, including genetic, epigenetic, metabolic, and environmental factors contributing to this malignant transformation [8].

The overall mortality rate for patients with MASLD is higher in comparison to a demographically matched general population [9]. Individuals with simple steatosis often have a better prognosis than those with MASH [10,11]. Prior research into MASH utilizing liver biopsy has been limited by participant numbers and outdated diagnostic criteria, limiting the applicability of these studies [10,11,12].

Liver biopsy is the definitive diagnostic method for grading and staging MASLD along with the NAFLD Activity Score (NAS), developed by the NASH Clinical Research Network (CRN), which evaluates steatosis, hepatocellular ballooning, and lobular inflammation excluding fibrosis [12].

This systematic review aimed to focus on the association between MASLD and the onset of hepatic cancer, considering the complexity of their relationship. By utilizing epidemiological studies, clinical trials, and molecular research, this study aimed to provide a comprehensive understanding of the risk factors and pathophysiological pathways involved along the way from MASLD to HCC.

## 2. Materials and Methods

### 2.1. Eligibility Criteria

The Preferred Reporting Items for Systematic Reviews and Meta-Analyses (PRISMA) reporting guidelines [13] were adhered to throughout the implementation of this systematic review.

The PECO (Population, Exposure, Comparator, Outcome) protocol was employed as the conceptual framework for formulating the research question and defining inclusion criteria. The population encapsulated individuals diagnosed with non-alcoholic fatty liver disease, including the entire spectrum from simple steatosis to non-alcoholic steatohepatitis. The exposure of interest was the presence of NAFLD/NASH, as diagnosed by histological, radiological, or biochemical assessment. The comparator (while not mandatory considering the observational nature of this review) included the absence of NAFLD/NASH or the general population without diagnosed liver disease. The primary outcome investigated was the incidence or prevalence of hepatocellular carcinoma in the context of NAFLD. Secondary outcomes included the identification of risk factors contributing to the progression of NAFLD to HCC. Studies were selected based on their ability to provide comparative data between the specified populations, with sufficient follow-up duration to assess the development of hepatic cancer.

### 2.2. Database Search Protocol

The search protocol for this review was designed to encompass a comprehensive retrieval of pertinent literature from seven distinct databases: PubMed, Embase, Scopus, Web of Science, Cochrane Library, CINAHL, and Google Scholar. Boolean operators (“AND”, “OR”, “NOT”) were adeptly applied in conjunction with medical subject headings (MeSH) and relevant keywords to refine and focus the search strategy, as elucidated in Table 1.

We carried out a literature search on seven different databases: PubMed, Embase, Scopus, Web of Science, Cochrane Library, CINAHL, and Google Scholar. We used search terms such as (“AND”, “OR”, “NOT”) in addition to Medical Subject Headings (MeSH) with relevant keywords, as illustrated in Table 2.

### 2.3. Variable Extraction Protocol

Following the identification of studies meeting the inclusion criteria, two reviewers independently embarked on the data extraction process using a standardized data extraction form specifically designed for the review. The extraction form had been previously piloted on a subset of included studies to fine-tune its applicability and ensure comprehensive data capture. The data extracted included bibliographic details (authors, year of publication, country of study), study characteristics (study design, sample size, duration of follow-up), participant demographics (age, sex, baseline characteristics), diagnostic criteria for non-alcoholic fatty liver disease (NAFLD) or non-alcoholic steatohepatitis (NASH), the definition of outcomes (incidence or prevalence of hepatocellular carcinoma (HCC)), and data necessary for the assessment of study quality and risk of bias (randomization, blinding, loss to follow-up).

In addition, information regarding the statistical analyses used, including measures of association (such as relative risks, odds ratios, hazard ratios), confidence intervals, and *p*-values, was meticulously recorded. Where available, data on potential confounders and effect modifiers were also extracted. Discrepancies between reviewers during the data extraction process were resolved through consensus or by consulting a third reviewer. To ensure consistency across reviewers, training sessions were conducted before the data extraction process commenced, and detailed instructions were provided regarding the use of the data extraction form. Upon the completion of data extraction, the information was thoroughly cross-checked between the reviewers to confirm accuracy.

### 2.4. Bias Assessment Protocol

The bias assessment protocol for this systematic review was structured around the Risk of Bias In Non-randomized Studies—of Exposures (ROBINS-E) tool [14], which is specifically designed for evaluating non-randomized studies of exposures. This tool was judiciously selected due to its comprehensive approach to appraising the risk of bias across multiple domains that may affect the validity of results within observational research.

### 2.5. GRADE Assessment Protocol

The assessment of the certainty of evidence in the studies selected for the review was conducted using the Grading of Recommendations Assessment, Development, and Evaluation (GRADE) approach [15], which was integrated with the findings from the bias assessment tools. Following the detailed evaluation of bias risk with the ROBINS-E tool [14], the GRADE methodology facilitated a systematic determination of the overall quality of evidence across outcomes.

## 3. Results

### 3.1. PRISMA Protocol Implementation

**Figure 1:**
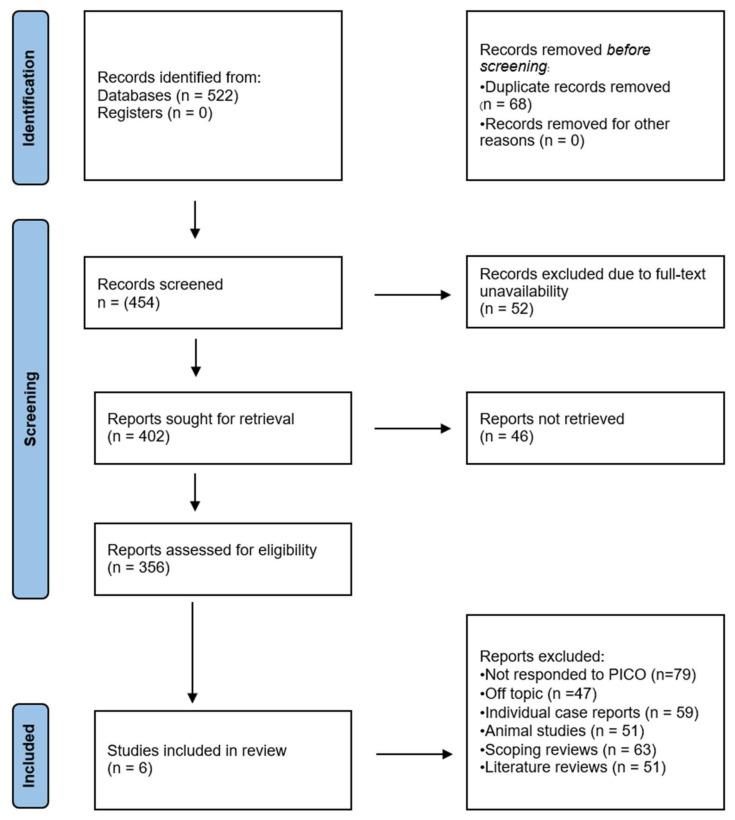
Different stages of the article selection process for this review.

Figure 1 shows the steps involved in selecting studies, starting with the initial 522 entries that were retrieved from the databases of choice. Nothing was obtained from the register. After 68 duplicate records were eliminated, screening was performed on the 454 records that remained. A total of 52 records were excluded due to access concerns, leaving 402 records for additional review. Another 46 records could not be obtained for a thorough evaluation. A thorough eligibility evaluation was conducted on the remaining 356 records. In this stage, 79 records did not meet the predetermined PICO criteria, and 47 records were eliminated for being unrelated to the research subject. Due to their inconsistency with the inclusion criteria, 51 narrative reviews, 51 animal studies, and 59 scoping reviews were also eliminated during this review phase. Six papers [16,17,18,19,20,21] were found to be appropriate for inclusion in the systematic review when these criteria were applied.

### 3.2. Bias Assessment Observations

**Figure 2:**
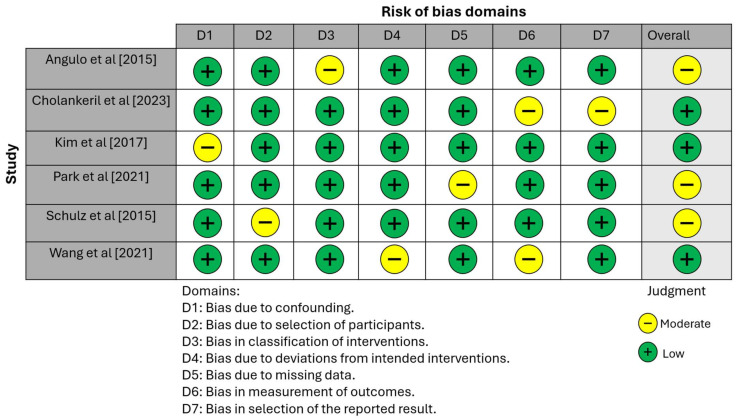
Bias assessment across different domains in the studies selected for the review [16,17,18,19,20,21].

As evident from the findings shown in Figure 2, the overall risk of bias varied across the studies. Angulo et al. [16] demonstrated a low risk of bias in most domains (D1, D2, D4, D5, D6, D7) but encountered a moderate risk in domain 3 (D3), leading to an overall moderate risk of bias. Cholankeril et al. [17] mostly showed a low risk of bias; however, the study had a moderate risk in the latter domains (D6 and D7), which resulted in an overall low risk of bias. Kim et al. [18] was the outlier with a moderate risk in the first domain (D1) and low risk across all other domains (D2 through D7), culminating in an overall low risk of bias for the study.

Park et al. [19] maintained a low risk of bias in most assessed areas (D1, D2, D3, D4, D6, D7) but faced a moderate risk in domain 5 (D5). This led to an overall moderate risk of bias. Schulz et al. [20] showed a low risk of bias across most domains except domain 2 (D2), where a moderate risk was identified. This resulted in an overall moderate risk of bias. Wang et al. [21] presented a low risk of bias in most domains (D1, D2, D3, D5, D7) but incurred a moderate risk in domains 4 and 6 (D4, D6), which established an overall low risk of bias.

### 3.3. Demographic Variables Assessed

Table 3 shows the studies included in the review and their observed conclusions. Angulo et al. [16] conducted a retrospective analysis of 619 subjects across the United States, Europe, and Thailand, with a substantial median follow-up duration of 12.6 years. Cholankeril et al. [17] employed a larger-scale study, utilizing data from the Veterans Administration hospitals, spanning from 2004 to 2018, and involved an impressive 202,319 subjects. This retrospective cohort study provided a robust dataset over an extended period, enabling a thorough investigation of the long-term impacts of MASLD. Kim et al. [18] analyzed data from a historical cohort of 25,947 individuals in Korea, with a follow-up exceeding 1 year and a median of 7.5 years. This study’s design allowed for a substantial examination of the temporal relationship between MASLD and various health outcomes within the Korean population.

Park et al. [19] tapped into the national health screening cohort in South Korea, following 8,120,674 adults up until December 2017. The vastness of this dataset offered a unique opportunity to recognize patterns and associations at a population level, contributing significantly to the understanding of MASLD’s broader implications. Schulz et al. [20] provided insights from a tertiary center, albeit the follow-up duration was not specified. This retrospective study’s smaller sample size of 120 subjects allowed for a more detailed individual analysis, which could offer valuable data on specific subgroups within the population. Wang et al. [21] derived their findings from a community-based cohort study in China, collecting data from June 2006 through October 2007 on 54,187 men. The study’s community-based approach and focus on a specific time frame offered a snapshot of the prevalence and implications of MASLD within that demographic.

### 3.4. Diagnostic and Analytical Parameters Observed

Angulo et al. [16] utilized laboratory and biopsy analyses for the diagnosis of MASLD, which are considered the gold standard for liver disease assessment. Their use of Cox proportional hazards regression for the main analysis suggests a focus on time-to-event data, which is crucial for understanding the progression of MASLD and its clinical outcomes. Cholankeril et al. [17], while not specifying the methods used for MASLD diagnosis, employed Landmark Fine–Gray competing risk models for their analysis. This approach is particularly useful in situations where multiple competing events are of interest, such as the development of various liver-related diseases or mortality, allowing for a detailed understanding of MASLD’s risks. Kim et al. [18] diagnosed MASLD using ultrasonographic detection of hepatic steatosis, a non-invasive and widely accessible method that can be applied to large-scale studies. They also used Cox proportional hazard regression for their analysis, which is robust at handling censored data and adjusting for confounding variables, thus providing a strong framework for assessing the impact of MASLD on health outcomes.

Park et al. [19] identified MASLD in their subjects using the fatty liver index (FLI), a non-invasive algorithm based on biomarkers and anthropometric measurements. This method allows for a broader application in large-scale epidemiological studies. Their analysis was conducted using Cox proportional hazards regression models, reinforcing the focus on the temporal dimension of disease progression. Schulz et al. [20] took a more direct approach by employing histological analysis of liver tissue for the diagnosis of MASLD, providing a high level of specificity in their findings. The statistical correlation applied in their analysis would have facilitated the investigation of relationships between histological findings and clinical outcomes. Wang et al. [21] used ultrasonography for MASLD diagnosis, a method that is practical for large, community-based studies due to its non-invasive nature and relative affordability. The Fine and Gray competing risk regression employed in their analysis indicates an effort to account for the presence of competing risks, a common issue in studies with long follow-up periods where participants may experience different types of events.

### 3.5. Key Findings Assessed

Angulo et al. [16] found that the stage of fibrosis in patients with MASLD was significantly associated with overall mortality, liver transplantation, and liver-related events. They observed that as the fibrosis stage increased, the risk of these outcomes also increased. Furthermore, they identified age, diabetes, current smoking, and statin use as significant predictors of these outcomes. Cholankeril et al. [17] reported that longitudinal changes in the FIB-4 index, a non-invasive marker of liver fibrosis, were associated with the risk of HCC and a composite endpoint of liver-related complications. They observed that a persistently high FIB-4 score significantly increased the risk of these outcomes.

Kim et al. [18] compared cancer incidence rates between MASLD and non-MASLD groups and found that the MASLD group had a higher rate of cancer. They established a strong association between MASLD and the development of HCC, colorectal cancer in males, and breast cancer in females. High scores on the MASLD fibrosis score and FIB-4 were also linked to an increased risk of all cancers, specifically HCC. Park et al. [19] discovered that MASLD was associated with an increased risk of biliary tract cancer (BTC), including cholangiocarcinoma and gallbladder cancer. They observed that the risk of BTC rose in conjunction with higher fatty liver index (FLI) scores. Additionally, the combination of MASLD with diabetes heightened the risk of BTC by 47%.

Schulz et al. [20] found that MASLD was more prevalent in patients with liver metastasis from colorectal cancer compared with those with other hepatic neoplasms. They also reported a higher prevalence of liver fibrosis in patients with hepatocellular carcinoma. However, they did not find a significant association of MASLD, MASH, or fibrosis with other risk factors in the tumors studied. Wang et al. [21] revealed that MASLD was associated with an increased risk of all cancers, thyroid cancer, and lung cancer. The association between MASLD and thyroid cancer was found to increase with elevated alanine aminotransferase (ALT) levels. Additionally, MASLD was linked to an increased risk of colorectal and lung cancer in smokers and to kidney cancer in non-diabetics.

### 3.6. Certainty Bias Assessment

Table 4 shows the GRADE assessment observations across the included studies. The risk of bias for all studies was reported as either low or low to moderate, indicating a reasonable level of confidence in the results. Inconsistency and indirectness were rated low across the studies, suggesting a direct and consistent measurement of outcomes and applicability to the research question. Imprecision was also rated as low, implying that the results were sufficiently precise to support a robust conclusion. There were no additional domains identified under ‘Others’ that would diminish the quality of evidence, such as publication bias or unexplained heterogeneity. Therefore, the overall certainty of the evidence for each study was considered high, supporting a strong recommendation for the observed findings within the context of the review.

## 4. Discussion

The studies analyzed in this review [16,17,18,19,20,21] present MASLD as a critical determinant of health, particularly in the context of liver fibrosis and its association with both hepatic and extra-hepatic malignancies. Angulo et al. [16] demonstrated a foundational link between the stage of fibrosis in MASLD with overall mortality and liver-related events, proposing a dose–response relationship.

This relationship was further supported by Cholankeril et al. [17], who provided evidence correlating elevations in the FIB-4 index with higher risks of hepatocellular carcinoma and cirrhosis. The emphasis on persistently high FIB-4 scores by Cholankeril et al. [17] aligns with the findings of Angulo et al. [16], both substantiating the prognostic utility of fibrosis staging.

In parallel, Kim et al. [18] extended the scope of NAFLD’s impact by demonstrating its role in the pathogenesis of diverse cancers, including HCC. Notably, their work suggested that the MASLD fibrosis score and FIB-4 are robust predictors of cancer risk, thus supporting the findings of Angulo et al. [16] and Cholankeril et al. [17] regarding the predictive value of fibrosis markers. The consistency across these studies [16,17,18] underscores a shared understanding of the importance of fibrosis evaluation in MASLD patients for risk stratification.

However, Park et al. [19] introduced an additional layer of complexity by identifying MASLD as a modifiable risk factor for biliary tract cancer (BTC), integrating the severity of MASLD and the co-morbid status of diabetes as compound risk amplifiers. This finding is particularly salient, as it diverges from the hepatic focus of previous studies and emphasizes the systemic implications of MASLD. Schulz et al. [20] further contributed to this systemic perspective, presenting evidence of an increased prevalence of hepatic neoplasms, such as metastatic colorectal cancer, among patients with MASLD. This outcome supports that MASLD’s morbidity extends beyond the liver itself, implicating it in a broader oncogenic process.

Wang et al. [21] synthesized these views by highlighting the multifactorial nature of cancer risk within the MASLD population, acknowledging the role of lifestyle and co-morbid conditions in risk stratification, and integrating extrinsic factors such as smoking and diabetes into the risk equation.

The heterogeneity of MASLD’s presentation was on a spectrum ranging from reversible steatosis to MASH. In Western cohorts, the yearly cumulative incidence of HCC in MASH-related cirrhosis is approximately 2.6% [22], whereas in Asian populations, a higher incidence of 11.3% has been reported [23].

Hamady et al. [24] presented hepatic steatosis as a secondary risk element for post-curative excision of colorectal cancer liver metastases (LMCC) and showed an association with poorer outcomes. Metastases often exhibit particular biological features, including a bilateral distribution, nodal involvement, and the presence of extraneous hepatic disease at the initial diagnosis. These factors, along with alterations in inflammatory cytokines and matrix remodeling enzymes, have been implicated in metastatic risk across various organ systems [25].

Pathophysiological shifts in steatosis and MASH have been linked to an up-regulation of specific signaling molecules, notably TGF-β and a subset of matrix metalloproteinases, which are thought to play roles in neoplastic pathways and the promotion of angiogenesis [25,26,27,28]. In contrast, there is research suggesting a lesser incidence of LMCC in those with MASLD, proposing steatosis may actually hinder LMCC formation [26,27,28,29,30], creating ambiguity regarding MASLD’s impact on LMCC development.

Within this study, the occurrence of malignant hepatic tumors was consistent with previously published data [31,32,33]. No significant disparities in the incidence of hepatic-steatosis-related neoplasms were observed when compared to the broader population (34.2% in the study cohort versus a general incidence of 20–30% [33]). Yet, a more detailed examination revealed a higher prevalence of hepatic steatosis within LMCC patients despite the level of concurrent liver fibrosis, indicating even minor steatosis could be an indicator of hepatic neoplasm development. Shared risk factors between steatosis and liver cancer include obesity, hyper-insulinemia, gastrointestinal disorders, and diabetes mellitus, with steatosis implicated in modifying hepatic microcirculation and inflammatory cytokines, potentially enhancing metastatic progression [8,34].

Chemotherapeutic interventions have been associated with increased rates of steatosis and steatohepatitis, reaching up to 92% in certain studies [8,35,36]. Chemotherapy-induced steatosis has been linked to augmented microcirculatory disturbances and a heightened vulnerability to complications such as micro-metastases [35]. The overlap between MASLD and colorectal liver metastases may be coincidental, given the high prevalence of hepatic steatosis in the general population, suggesting a lack of a definitive causal relationship between these conditions.

### 4.1. Limitations

The limitations of this study were influenced by the generalizability of the findings. Primarily, the diagnostic criteria for MASLD and the assessment of liver fibrosis relied on a range of modalities, from invasive liver biopsies to non-invasive imaging and biochemical scoring systems. This discrepancy in diagnostic methodologies could have led to misclassification biases, potentially skewing the reported prevalence and incidence rates of fibrosis and cancer. The reliance on surrogate markers of fibrosis, such as the FIB-4 index and the MASLD fibrosis score, while practical, may not have captured liver pathology as accurately as histological examinations. Additionally, data on the natural history of MASLD in diverse populations were scarce. Most studies were conducted in Western or Asian cohorts, which may not reflect the disease progression and associated cancer risks in other ethnic and geographical populations. This limits the extrapolation of the study findings to a global context and highlights the need for research in a wider array of demographic groups.

The influence of confounding factors such as alcohol consumption, medication use, and other concurrent liver diseases was not uniformly assessed across studies. These variables could have independently influenced the risk of developing hepatic and extra-hepatic cancers, thereby confounding the relationship between MASLD and malignancy. While associations were robustly demonstrated, the observational study designs do not permit definitive conclusions regarding causality between MASLD, liver fibrosis, and cancer development.

### 4.2. Recommendations

Several recommendations emerge that can guide clinical practice and health policy. First and foremost, it is recommended that patients with MASLD should be stratified according to their stage of liver fibrosis, as this is an independent predictor of mortality and liver-related events. Regular monitoring using non-invasive tools such as the FIB-4 index or the MASLD fibrosis score should be implemented to track fibrosis progression. Secondly, given the correlation between fibrosis severity and adverse outcomes, it is advisable to adopt a risk-based approach to the management of MASLD patients. Those exhibiting higher stages of fibrosis or longitudinal increases in fibrosis scores should be considered for more aggressive therapeutic interventions to mitigate the risk of progression to HCC, cirrhosis, and other forms of advanced liver disease.

Furthermore, the association of MASLD with both hepatic and extra-hepatic cancers suggests that screening protocols for cancers such as colorectal in males, breast in females, and biliary tract cancers may be warranted in patients with significant MASLD, especially those with high fibrosis scores or diabetes. This recommendation is consistent with the established practice of individualized cancer screening based on risk factors, and MASLD should be recognized as one of these factors.

Moreover, recognizing the potential role of lifestyle factors in modifying disease risk, it is advisable to integrate lifestyle interventions as a core component of MASLD management. Additionally, the findings underscore the need for a comprehensive approach to the management of MASLD that encompasses not only the hepatic manifestations of the disease but also its systemic implications. Multi-disciplinary teams involving hepatologists, oncologists, endocrinologists, and primary care providers should collaborate to optimize the overall health outcomes for patients with MASLD. This holistic approach should be supported by patient education on the risks associated with MASLD and the importance of lifestyle modifications and regular follow-ups to effectively manage the disease and its complications.

## 5. Conclusions

The study illuminated the substantial role of MASLD as a significant health concern, with outcomes extending beyond hepatic morbidity. The findings highlighted the prognostic significance of liver fibrosis within the MASLD spectrum, affirming it as an independent predictive factor of mortality and liver-related events. It was determined that the progression of fibrosis correlated positively with the risk of adverse outcomes, establishing a quantifiable relationship between the extent of liver damage and the likelihood of harmful health events. Supported by recent studies on fibrosis markers, this risk appeared to be cumulative over time, particularly in those individuals who exhibited persistent elevations in their fibrosis scores [17].

Moreover, the progression of fibrosis, as measured by recognized indices, was found to be a predictor of heightened risk for the onset of hepatocellular carcinoma, highlighting the need for interventions to better identify individuals at risk [37]. Concurrently, the study identified MASLD as a factor implicated in the etiology of a range of neoplasms beyond the liver, suggesting a systemic impact of the disease. In addition to the direct implications of MASLD and fibrosis on hepatic and extra-hepatic cancer risk, the study illuminated the influence of co-morbid conditions, such as diabetes, and lifestyle factors, including smoking, on the disease trajectory. These findings suggest that the interplay between MASLD and such modifiers could further refine the risk stratification for patients, pointing toward a multifaceted approach to risk assessment.

## Figures and Tables

**Table 1:** The different selection criteria that were employed for this review.

Criteria	Inclusion	Exclusion
Study Design	Peer-reviewed observational studies, clinical trials, and cohort studies	Reviews, editorials, commentaries, and case reports
Participants	Subjects diagnosed with non-alcoholic fatty liver disease (NAFLD) or non-alcoholic steatohepatitis (NASH)	Subjects with alcohol-related liver disease, viral hepatitis, or other specific causes of liver disease
Interventions/Exposures	Presence of NAFLD/NASH confirmed by biopsy, imaging, or clinical/biochemical criteria	Studies lacking clear diagnostic criteria for NAFLD/NASH
Outcomes	Incidence or prevalence of hepatocellular carcinoma (HCC)	Studies not reporting on HCC or only reporting surrogate outcomes

**Table 2:** Inclusion and exclusion criteria devised for the review.

Database	Search String
PubMed	(“Non-alcoholic Fatty Liver Disease”[MeSH Terms] OR NAFLD[Text Word] OR NASH[Text Word]) AND (“Carcinoma, Hepatocellular”[MeSH Terms] OR HCC[Text Word] OR “liver cancer”[Text Word]) AND (“Risk Factors”[MeSH Terms] OR risk[Text Word])
Embase	(‘non-alcoholic fatty liver disease’/exp OR NAFLD OR NASH) AND (‘hepatocellular carcinoma’/exp OR HCC OR ‘liver cancer’) AND (‘risk factor’/exp OR risk)
Scopus	(TITLE-ABS-KEY (“Non-alcoholic Fatty Liver Disease” OR NAFLD OR NASH)) AND (TITLE-ABS-KEY (“Hepatocellular Carcinoma” OR HCC)) AND (TITLE-ABS-KEY (risk OR epidemiology))
Web of Science	(TI=(“Non-alcoholic Fatty Liver Disease” OR NAFLD OR NASH)) AND (TI=(“Hepatocellular Carcinoma” OR HCC)) AND (TI=(risk OR epidemiology))
Cochrane Library	(“Non-alcoholic Fatty Liver Disease”:ti,ab,kw OR NAFLD:ti,ab,kw OR NASH:ti,ab,kw) AND (“Hepatocellular Carcinoma”:ti,ab,kw OR HCC:ti,ab,kw) AND (risk:ti,ab,kw OR epidemiology:ti,ab,kw)
CINAHL	(MH “Non-alcoholic Fatty Liver Disease” OR NAFLD OR NASH) AND (MH “Carcinoma, Hepatocellular” OR HCC OR “liver cancer”) AND (MH “Risk Factors” OR risk OR epidemiology)
Google Scholar	(“Non-alcoholic Fatty Liver Disease” OR NAFLD OR NASH) AND (“Hepatocellular Carcinoma” OR HCC OR “liver cancer”) AND (risk OR epidemiology)

**Table 3:** Search strings utilized across the different databases.

Study ID	Study Design	Location	Follow-Up Duration	Subjects	NAFLD Diagnosis Method	Main Analysis Method	Key Findings	Inference Observed
Angulo et al. [15]	Retrospective analysis	United States, Europe, Thailand	Median 12.6 years	619	Laboratory and biopsy analyses	Cox proportional hazards regression	Fibrosis stage was significantly associated with overall mortality, liver transplantation, and liver-related events. Higher fibrosis stages correlated with increased risk. Age, diabetes, current smoking, and statin use were significant predictors of outcomes.	Fibrosis stage is an independent predictor of mortality and liver-related events in NAFLD, with higher stages indicating greater risk.
Cholankeril et al. [16]	Retrospective cohort study	Veterans Administration hospitals	1 January 2004–31 December 2018	202,319	Not specified	Landmark Fine–Gray competing risks models	Longitudinal changes in FIB-4 associated with risk of HCC and a composite endpoint. Persistently high FIB-4 significantly increased risk.	Longitudinal increases in FIB-4 were associated with higher risks of HCC and cirrhosis in NAFLD patients, especially with persistent high FIB-4.
Kim et al. [17]	Historical cohort study	Korea	>1 year (median 7.5 years)	25,947	Ultrasonographic detection of hepatic steatosis	Cox proportional hazards regression	NAFLD group had a higher cancer incidence rate compared to non-NAFLD group. NAFLD strongly associated with HCC, colorectal cancer in males, and breast cancer in females. High fibrosis score and FIB-4 scores linked to all cancers and HCC.	NAFLD was associated with the development of certain types of cancer, with high fibrosis scores and FIB-4 scores indicating a strong association.
Park et al. [18]	National health screening cohort	South Korea	Followed up until December 2017	8,120,674 adults	Fatty liver index (FLI)	Cox proportional hazards regression models	NAFLD was associated with an increased risk of BTC, including cholangiocarcinoma and gallbladder cancer. The risk of BTC increased with higher FLI scores. NAFLD combined with diabetes increased the risk of BTC by 47%.	NAFLD was identified as a potentially modifiable risk factor for BTC, with the risk increasing with NAFLD severity and presence of diabetes.
Schulz et al. [19]	Retrospective study	Tertiary center	Not specified	120	Histological analysis of liver tissue	Histological analysis and statistical correlation	NAFLD was more prevalent in patients with liver metastasis of colorectal cancer than with other hepatic neoplasms. Higher prevalence of liver fibrosis in patients with hepatocellular carcinoma. No significant association of NAFLD, NASH, or fibrosis with other risk factors in tumors studied.	NAFLD prevalence was comparable with the general population but showed a higher association with certain types of hepatic neoplasms, such as metastatic colorectal cancer.
Wang et al. [20]	Community-based cohort study	China	June 2006–October 2007	54,187 men	Ultrasonography	Fine and Gray competing risk regression	NAFLD was associated with an increased risk of all cancers, thyroid cancer, and lung cancer. The association of NAFLD with thyroid cancer increased with ALT levels. NAFLD increased the risk of colorectal and lung cancer in smokers and kidney cancer in non-diabetics.	NAFLD is linked to a higher risk of various cancers, with certain lifestyle factors (smoking) or comorbidities (diabetes) modifying the risk.

Abbreviations: Non-alcoholic fatty liver disease, NAFLD; hepatocellular carcinoma, HCC, biliary tract cancer, BTC; alanine aminotransferase, ALT; fibrosis index based on four factors, FIB-4.

**Table 4:** GRADE assessment observations.

Study Design	Number of Studies	Observed Common Finding	Risk of Bias	Inconsistency	Indirectness	Imprecision	Others	Certainty
Cohort studies (retrospective and prospective)	6	Fibrosis stage and FIB-4 index are predictors of mortality, liver-related events, and certain types of cancer in patients with NAFLD	Low to moderate	Low	Low	Low	None	High

Abbreviations: Non-alcoholic fatty liver disease, NAFLD; fibrosis index based on four factors; FIB-4.

## Data Availability

No new data were created.
